# Baseline Anti-SARS-CoV-2 IgG and Protection from Symptomatic Infection: Post Hoc Analysis of the SCTV01E Phase 3 Randomized Trial

**DOI:** 10.3390/vaccines13090984

**Published:** 2025-09-19

**Authors:** Lixin Yan, Jiang Yi, Dongfang Liu, Jian Li, Adam Abdul Hakeem Baidoo, Liangzhi Xie

**Affiliations:** 1 Beijing Engineering Research Center of Protein and Antibody, Sinocelltech Ltd., No. 31 Kechuang 7th Street, BDA, Beijing 100176, China; lixin_yan@sinocelltech.com (L.Y.); jiang_yi@sinocelltech.com (J.Y.); dongfang_liu@sinocelltech.com (D.L.); jian_li@sinocelltech.com (J.L.); baidooadam@sinocelltech.com (A.A.H.B.); 2 Beijing Key Laboratory of Monoclonal Antibody Research and Development, Sino Biological Inc., Beijing 100176, China; 3 Cell Culture Engineering Center, Chinese Academy of Medical Sciences & Peking Union Medical College, Beijing 100005, China

**Keywords:** COVID-19, anti-SARS-CoV-2 IgG antibody, relative protection

## Abstract

**Background:** Following SARS-CoV-2 infection, the necessity of vaccination after natural infection remains uncertain. However, many asymptomatic individuals who test negative virologically may nevertheless receive vaccination without being aware of their prior infection. Investigating the implications for vaccine safety and efficacy is crucial. **Methods:** We analyzed the daily fluctuations in anti-SARS-CoV-2 IgG antibody levels during the enrollment period of a phase 3 randomized, double-blinded, placebo-controlled clinical trial of a tetravalent COVID-19 protein vaccine, SCTV01E. Additionally, we investigated the relationship between baseline IgG levels and their protection against COVID-19 in participants who received placebo. **Results:** The daily enrolled participants with different baseline IgG levels (<338 BAU/mL, 338–1000 BAU/mL, >1000 BAU/mL) showed dynamic changes with the enrollment date. Among participants with baseline IgG levels < 338 BAU/mL, vaccination conferred a relative protective efficacy of 69.15% (95% CI: 51.14–80.52%) against symptomatic SARS-CoV-2 infection compared with the control group. Conversely, in those with higher baseline IgG levels (≥338 BAU/mL), vaccination did not confer additional benefit. In the placebo group, the relative protection in participants with baseline IgG levels ≥ 338 BAU/mL was 93.79% (87.60%, 96.89%) compared to that of those with baseline IgG levels < 338 BAU/mL. The safety profile of SCTV01E in participants with baseline IgG ≥ 338 BAU/mL was comparable to that in participants with <338 BAU/mL, with favorable safety profiles. **Conclusions:** During the SCTV01E phase 3 clinical trial, an anti-SARS-CoV-2 IgG antibody IgG level of 338 BAU/mL was suitable for screening individuals in the early phase post-infection alongside virological tests. Vaccinating the infected population was safe and did not compromise efficacy. Clinical Trial: NCT05308576.

## 1. Introduction

Vaccination emerged as a crucial modality in the global fight to reduce the transmission, morbidity, mortality, and overall burden of Coronavirus disease 2019 (COVID-19) [1,2]. As of December 2024, over 13 billion COVID-19 vaccines have been used [1]. Although previous phase III trials have consistently demonstrated the efficacy and safety of vaccines in preventing symptomatic SARS-CoV-2 infection, those trials primarily excluded participants with a documented history of SARS-CoV-2 infection to minimize potential confounding effects on efficacy and safety assessments [3,4,5]. However, it is important to note that individuals who were asymptomatic and presented with negative virological results were likely included. As a result, there are uncertainties about the safety and efficacy of vaccination in early phase post-infected individuals who are vaccinated without knowing their infection status. Moreover, in the event that a vaccine clinical trial is conducted during a pandemic, it is imperative to ensure the scientific validity and plausibility of the results obtained from such a trial. It is therefore essential to minimize, as far as possible, the inclusion of individuals in the early post-infection phase.

Besides virological tests, one key immunological indicator of SARS-CoV-2 exposure history is the anti-SARS-CoV-2 IgG antibody, which is detectable from approximately 11 days post-infection and gradually declines within six months post-infection [6,7,8,9]. Previous studies have identified a correlation between anti-SARS-CoV-2 IgG antibody levels and protection against infection. However, no definitive IgG antibody threshold has been established to distinguish early phase post-infection status [10,11]. Following the surge of SARS-CoV-2 infections after gradually lifting the stringent COVID-19 restrictions in China on 8 December 2022, the overall infection rate was estimated to be 87.54% by 30 January 2023 [12,13]. As a result, a substantial proportion of individuals may have developed high IgG antibody levels.

SCTV01E is a tetravalent COVID-19 protein vaccine containing spike protein ectodomain of the Alpha, Beta, Delta, and Omicron BA.1 variants. SCTV01E received Emergency Use Authorization in China and the United Arab Emirates. The phase 3 clinical trial of SCTV01E was conducted immediately after China’s policy of strict COVID-19 control was lifted [14]. At that time, many individuals in early phase post-infection were enrolled in the trial. To obtain scientific and reliable data on the efficacy and safety of SCTV01E, based on the findings from prior studies of SCTV01E, an IgG level of ≥338 BAU/mL was set to indicate the early phase post-SARS-CoV-2 infection [14].

This report was a post hoc analysis of the SCTV01E phase 3 randomized trial conducted to evaluate two key objectives: first, how baseline anti-SARS-CoV-2 IgG levels relate to subsequent symptomatic infection risk and vaccine benefit; and second, whether SCTV01E was efficacious and safe in individuals during this early post-infection phase.

## 2. Materials and Methods

### 2.1. Study Design and Participants

The data used for this post hoc analysis were from a phase 3 study, which was a randomized, double-blind, placebo-controlled clinical trial to assess the efficacy and safety of SCTV01E in preventing SARS-CoV-2 infection [14]. The trial was conducted across Guizhou, Sichuan, and Hunan Province CDC centers in China, and provincial ethics approvals were obtained. Baseline blood was collected prior to randomization for IgG and nasopharyngeal/oropharyngeal swab for PCR testing. Subjects were assigned in a 1:1 ratio to receive either the investigational vaccine or placebo. The trial enrolled participants aged 18 years or older who had received a previous COVID-19 vaccine 6 to 24 months prior or had a history of SARS-CoV-2 infection within the last 6 months. In total, 9175 participants had baseline IgG results, of whom 4584 were assigned to the placebo group and 4591 to the SCTV01E group. Of these, 5891 (58.5%) had baseline IgG ≥ 338 BAU/mL, while 3284 (41.5%) had IgG < 338 BAU/mL. The participants had a median age of 51 years and a median BMI of 23.9 kg/m^2^. A total of 14.6% of enrolled individuals were PCR-positive at baseline. The median interval between the last vaccination and administration of SCTV01E or placebo was 13 months. Baseline demographic characteristics were well balanced across the subgroups, with detailed data provided in Table S1, and the detailed study design was previously published [14].

An analysis was conducted on the daily fluctuations in IgG levels during the enrollment period of a phase 3 randomized, double-blind, placebo-controlled clinical trial of a tetravalent protein vaccine against SARS-CoV-2. The vaccine is referred to as SCTV01E. Participants in the placebo group were chosen for analysis to investigate the relationship between protection against symptomatic SARS-CoV-2 infection and baseline IgG levels. A stratified protection analysis against symptomatic SARS-CoV-2 infection across different groups was performed using a baseline IgG level of 338 BAU/mL as a cutoff. Adverse events related to SCTV01E were analyzed based on baseline anti-SARS-CoV-2 IgG concentrations (<338 BAU/mL, ≥338 BAU/mL) and baseline virological results (PCR positive, PCR negative). Furthermore, the safety of participants aged ≥60 years was also specifically evaluated.

### 2.2. Statistical Analysis

The analysis included randomized participants who completed the planned vaccination schedule and had valid baseline IgG levels without major protocol deviations. Statistical analyses were performed using SAS version 9.4 (SAS Institute Inc., Cary, NC, USA).

Protection against symptomatic SARS-CoV-2 infection was calculated as 1-HR, with HR derived from the Cox regression model. If the calculated value of protection against symptomatic SARS-CoV-2 infection (a vs. b) was less than zero, it was adjusted to minus (b vs. a) to keep the range within [−1, 1]. Fisher’s exact test was used to assess inter-group differences in the incidence of adverse events.

## 3. Results

### 3.1. Proportion of Participants with Different Baseline IgG Levels with the Enrollment Date

**Figure 1:**
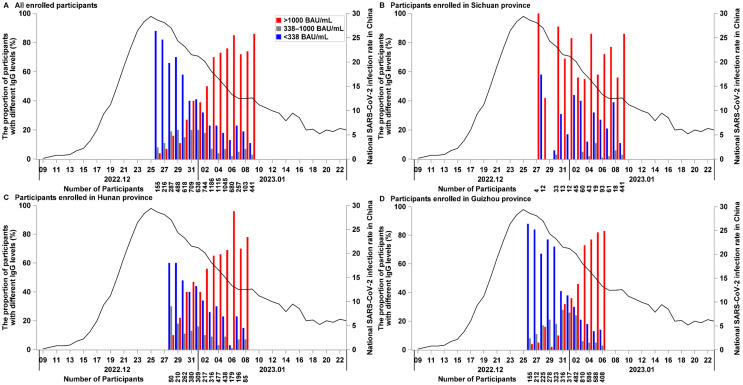
Changes in the proportion of baseline anti-SARS-CoV-2 IgG antibody levels across non-immunogenic subgroups based on enrollment dates. (**A**) All enrolled participants; participants in (**B**) Sichuan Province, (**C**) Hunan Province, and (**D**) Guizhou Province. The black curve represents changes in China’s national SARS-CoV-2 infection rate over time [12].

The study was initiated during the period with the maximum national SARS-CoV-2 infection rate in China, as indicated by data from the Chinese Center for Disease Control and Prevention (CDC) [12], the black curve depicted in Figure 1 represents changes in China’s national SARS-CoV-2 infection rate over time. The baseline distribution of IgG levels among participants enrolled daily is depicted in Figure 1. As illustrated in Figure 1A, the proportion of participants with low antibody levels (<338 BAU/mL) gradually decreased over time, while the proportion with high antibody levels (>1000 BAU/mL) progressively increased. Notably, participants’ antibody levels peaked later than reported SARS-CoV-2 infection cases, which is consistent with the expected antibody kinetics.

As illustrated in Figure 1C,D, the trends observed in Hunan Province and Guizhou Province were consistent with the national trajectory [12]. However, in Sichuan Province (see Figure 1B), the proportion of participants with antibody levels greater than 1000 BAU/mL remained consistently high from the first day of enrollment. The earlier peak observed in Sichuan may be due to differences in how quickly reopening policies were implemented. Sichuan Province reopened before Guizhou and Hunan. This time difference aligns with the IgG dynamics illustrated in Figure 1.

### 3.2. Relationship Between Baseline Anti-SARS-CoV-2 IgG and Relative Protection Against Symptomatic SARS-CoV-2 Infection

Among the 4584 participants in the placebo group, 84 cases of symptomatic SARS-CoV-2 infections were reported. These participants were categorized into eight groups based on their baseline antibody levels, which allowed us to calculate the annual incidence rate of events and the corresponding relative protection for each group. The control group, used for calculating relative protection, consisted of participants with baseline IgG levels below 26 BAU/mL (twice the lower limit of quantification, 13 BAU/mL). Subsequent groups were defined using multiples of 26 BAU/mL as thresholds. The interval width of these antibody level ranges increased progressively across groups, with each group maintaining a sample size sufficient for robust statistical analysis.

**Figure 2:**
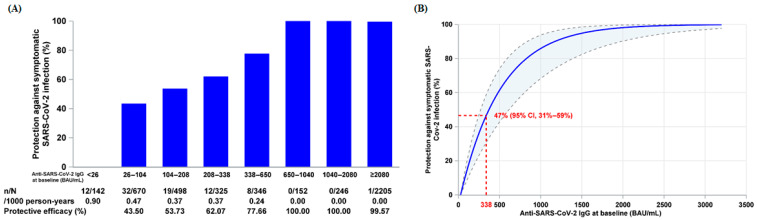
Relationship between baseline anti-SARS-CoV-2 IgG antibody levels and protection against symptomatic SARS-CoV-2 infection. (**A**) Bars represent the relative protection of each group compared to the control group, calculated as 1 minus the hazard ratio (HR) obtained from a Cox regression model. (**B**) The curve illustrates the protection at varying baseline IgG levels relative to a 26 BAU/mL baseline value (*y* = 1 − *e*^−0.002009(^*^x^*^ − 26)^), with the shaded area representing the 95% confidence interval for relative protection against symptomatic SARS-CoV-2 infection (*y* = 1 − *e*^−0.002009(^*^x^*^ − 26) ± 1.96 × 0.0004240(^*^x^*^ − 26)^). Parameters were estimated using a Cox regression model, with baseline anti-SARS-CoV-2 IgG antibody concentration as the explanatory variable.

As shown in Figure 2A, the annual incidence rate of events progressively decreased with increasing baseline antibody levels. When baseline IgG levels were ≥338 BAU/mL, the relative protection in each group surpassed 70%, signifying adequate protection against infection. Furthermore, 75 of the 84 infection cases occurred in participants with baseline IgG levels < 338 BAU/mL. This cutoff point of 338 BAU/mL effectively excludes individuals at lower risk of infection while capturing the majority of infection cases, validating its suitability as an appropriate threshold.

The relationship between baseline IgG levels and infection risk in the placebo group was further analyzed using the Cox proportional hazard model, with the relative protection curve concerning baseline antibodies at 26 BAU/mL shown in Figure 2B. The analysis revealed that at lower antibody levels, relative protection increased significantly with rising baseline antibody levels. However, after reaching a high level of relative protection, additional increases in baseline antibody levels resulted in a slower rate of improvement. Overall, the risk of infection decreases with increasing antibody levels. When baseline IgG levels reached 338 BAU/mL, the relative protection was 47% (95% CI, 31–59%) compared to those with baseline IgG antibody levels below 26 BAU/mL.

### 3.3. Stratified Analysis of Relative Protection Against Symptomatic SARS-CoV-2 Infection by Baseline IgG Levels

**Figure 3:**
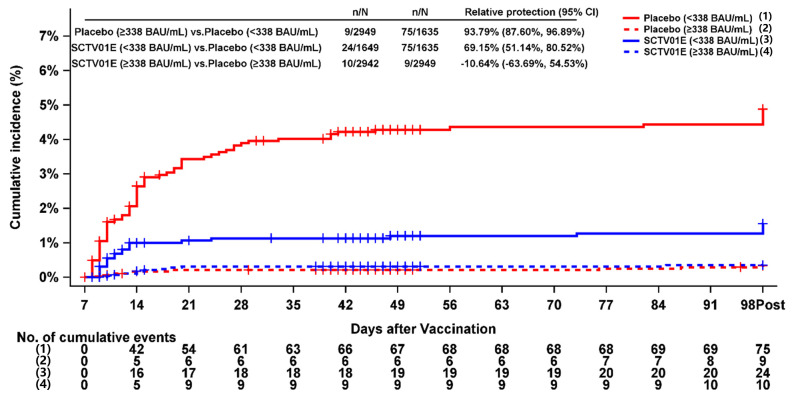
Cumulative incidence of symptomatic SARS-CoV-2 infection across different subgroups. Cross marks indicate censored participants. The value of 338 represents participants with a baseline IgG concentration of 338 BAU/mL.

As shown in Figure 3, among participants with baseline IgG levels < 338 BAU/mL, the relative protection observed in the SCTV01E group compared to the placebo group was 69.15% (51.14%, 80.52%), effectively demonstrating the vaccine’s protective effect in this population. Conversely, for participants with baseline IgG levels ≥ 338 BAU/mL, the relative protection of the SCTV01E group compared to the placebo group fell below zero, indicating no additional efficacious benefits from vaccination.

In the placebo group, the relative protection for participants with baseline IgG levels of ≥338 BAU/mL was 93.79% (95% CI, 87.60–96.89%) compared to those with baseline IgG levels of <338 BAU/mL, as shown in Figure 3.

### 3.4. Safety

**Figure 4:**
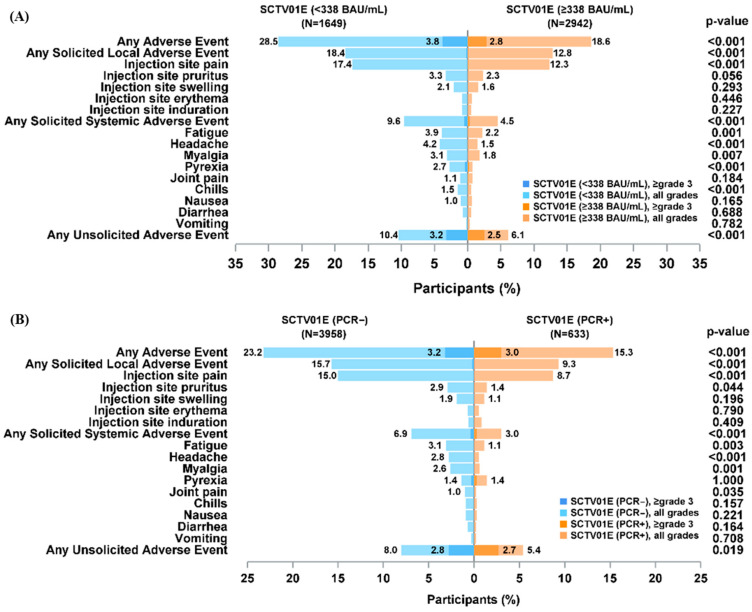
The incidence of adverse events is stratified by anti-SARS-CoV-2 IgG antibody levels (Panel (**A**)) and PCR results (Panel (**B**)). Events classified as Grade ≥ 3 are defined as severe or higher-grade adverse events.

A significant difference in adverse events incidence was noted between the two stratified groups based on baseline IgG levels (*p* < 0.001) (Figure 4A). The incidence of AEs was 18.6% in the baseline anti-SARS-CoV-2 IgG ≥ 338 BAU/mL subgroup and 28.5% in the baseline IgG < 338 BAU/mL subgroup. Within seven days after vaccination, both solicited local AEs and systemic AEs occurred less frequently in the baseline IgG ≥ 338 BAU/mL subgroup compared to the baseline IgG < 338 BAU/mL subgroup (*p* < 0.001). For the two most commonly solicited AEs, injection site pain and fatigue, the incidence in the baseline IgG ≥ 338 BAU/mL subgroup was lower than that in the baseline IgG < 338 BAU/mL subgroup (*p* < 0.001). These findings indicate that pre-existing humoral immunity may mitigate post-vaccination reactogenicity, suggesting that individuals with higher baseline IgG levels are less prone to developing local and systemic AEs. In the subgroup analysis according to baseline PCR status, the safety profile of participants with positive or negative baseline PCR was comparable after SCTV01E vaccination. Participants with positive baseline PCR were also safe after SCTV01E vaccination (Figure 4B).

The vaccination of SCTV01E was found to be safe for older adults (aged ≥60 years) (Figure S1). Specifically, among older adults, the incidence of AEs was 15.9% in participants with IgG ≥ 338 BAU/mL, which is comparable to 17.6% in those with anti-SARS-CoV-2 IgG < 338 BAU/mL (Figure S1). Similarly, among older adults, the AE incidence in the PCR-positive subgroup was 15.3%, lower than the 16.9% observed in the PCR-negative subgroup (Figure S1B).

## 4. Discussion

Following the lifting of COVID-19 restrictions in mainland China, the number of infections surged sharply. Between 8 December 2022, and 23 January 2023, the daily infection rate peaked at 29.2% on 25 December 2022. Subsequently, it declined gradually, dropping to 5.5% by 23 January 2023 [12]. As of 30 January 2023, the estimated overall infection rate reached 87.54% [13]. The enrollment period for the phase 3 study, which spanned from 26 December 2022, to 15 January 2023, coincided with the peak of this epidemic wave, during which Omicron was the predominant variant [12]. The asymptomatic infection rate for Omicron was 41.72%, according to epidemiological data from Quanzhou, Fujian Province, China [15]. The proportion of participants with baseline anti-SARS-CoV-2 IgG ≥ 338 BAU/mL was 58.5% in this phase 3 study. Despite the exclusion of individuals with SARS-CoV-2 infections occurring within the previous six months, a considerable number of asymptomatic participants in the early phase post-SARS-CoV-2 infection, some of whom exhibited negative virological results, were ultimately enrolled during the pandemic.

Notably, the phase 3 study showed that, as the overall infection rate increased, the proportion of participants with baseline IgG levels < 338 BAU/mL gradually decreased, while the proportion with IgG levels > 1000 BAU/mL reached approximately 80% on 3 January 2023. This indicates that many enrolled participants were in the early phase after infection, and baseline IgG antibody levels increased over time. Previous studies have shown a strong positive association between IgG levels and protective efficacy, and detectable antibodies confers protection against reinfection for at least six months [10,11].

The relationship between baseline IgG levels and relative protection was evaluated solely in the placebo group to isolate the effects of the vaccine. Participants with baseline IgG levels ≥ 338 BAU/mL exhibited over 70% relative protection compared to those with baseline IgG levels < 26 BAU/mL. Specifically, at a baseline IgG level of 338 BAU/mL, the relative protection in the placebo group was 47% (31%, 59%). Furthermore, when baseline IgG levels were ≥338 BAU/mL, the relative protection of SCTV01E fell below zero, with low infection rates observed in both the SCTV01E and placebo groups. Conversely, for participants with baseline IgG levels < 338 BAU/mL, SCTV01E showed 69.4% (95% CI, 50.6–81.0%) efficacy. The findings indicate that SCTV01E exhibited significant protective effects in participants with low baseline IgG levels. However, it did not provide additional protection in participants with high baseline IgG levels. This suggests that high baseline IgG levels offer protection against SARS-CoV-2 infection, thereby reducing the benefits of vaccination.

In previous studies, SCTV01E and related vaccines showed a favorable safety profile [14,16,17,18,19], with safety outcomes being better in participants aged 60 and older, where the incidence of adverse events was lower than in those aged 18–59 [12]. This post hoc analysis demonstrated that the incidence of adverse events (AEs) was lower in the anti-SARS-CoV-2 IgG ≥ 338 BAU/mL subgroup compared to the anti-SARS-CoV-2 IgG < 338 BAU/mL subgroup. These results align with the subgroup analysis based on baseline PCR status, suggesting that individuals in the early phase post-SARS-CoV-2 infection can safely receive SCTV01E. Furthermore, age-stratified analyses demonstrated that SCTV01E was safe for older adults (aged ≥60 years) post-SARS-CoV-2 infection.

The trial has two limitations: First, post hoc analysis was conducted using data from Chinese participants, which may not generalize beyond Chinese population. Second, the IgG assay is not calibrated to international units across platforms, which may limit the generalizability of the assay platform used.

## 5. Conclusions

The baseline anti-SARS-CoV-2 IgG antibody level of 338 BAU/mL used in this study serves as an appropriate cutoff value for distinguishing the early phase post-infection status of participants, thereby enhancing the authenticity and reliability of the results from the phase 3 vaccine clinical trial. The procedure remains safe when an individual is vaccinated with SCTV01E without knowing their infection status. However, the vaccine does not provide additional protection against symptomatic SARS-CoV-2 infection beyond the inherent anti-SARS-CoV-2 protection antibodies.

## Data Availability

The authors declare that the data supporting the findings of this study are available in the main manuscript or the Supplementary Material. Correspondence and requests for materials should be addressed to L.X. (lx@sinocelltech.com).

## Supplementary Materials

The following supporting information can be downloaded at: https://www.mdpi.com/article/10.3390/vaccines13090984/s1, Table S1: Demographic Characteristics of Participants; Figure S1: The incidence of adverse events is stratified by anti-SARS-CoV-2 IgG antibody levels and age (Panel A), PCR results and age (Panel B). Events classified as Grade ≥ 3 are defined as severe or higher-grade adverse events.
