# Supplementary material for: Baseline Anti-SARS-CoV-2 IgG and Protection from Symptomatic Infection: Post Hoc Analysis of the SCTV01E Phase 3 Randomized Trial
Source: Vaccines (Basel). 2025 Sep 19;13(9):984. doi: 10.3390/vaccines13090984 (PMC12474267; doi:10.3390/vaccines13090984)
Supplement: Supplementary file 1 [file vaccines-13-00984-s001.zip › vaccines-3846326-supplementary.pdf]

Tabel S1. Demographic Characteristics of Participants

	Placebo			SCTV01E			
	<338 BAU/mL (N=1635)	≥338 BAU/mL (N=2949)	Total (N=4584)	<338 BAU/mL (N=1649)	≥338 BAU/mL (N=2942)	Total (N=4591)	All (N=9175)
Age (Years)							
N (Missing)	1635 (0)	2949 (0)	4584 (0)	1649 (0)	2942 (0)	4591 (0)	9175 (0)
Mean (SD)	51.2 (15.20)	48.4 (14.47)	49.4 (14.79)	51.7 (14.58)	48.3 (14.79)	49.5 (14.80)	49.5 (14.80)
Median	53.0	50.0	51.0	53.0	50.0	51.0	51.0
Min, Max	18, 88	18, 87	18, 88	18, 92	18, 95	18, 95	18, 95
Age subgroups randomization, n (%)							
18-59Years	1143 (69.9)	2322 (78.7)	3465 (75.6)	1158 (70.2)	2308 (78.5)	3466 (75.5)	6931 (75.5)
≥60Years	492 (30.1)	627 (21.3)	1119 (24.4)	491 (29.8)	634 (21.5)	1125 (24.5)	2244 (24.5)
Sex, n (%)							
Male	999 (61.1)	1611 (54.6)	2610 (56.9)	1010 (61.2)	1640 (55.7)	2650 (57.7)	5260 (57.3)
Female	636 (38.9)	1338 (45.4)	1974 (43.1)	639 (38.8)	1302 (44.3)	1941 (42.3)	3915 (42.7)
BMI (kg/m^2)							
N (Missing)	1634 (1)	2949 (0)	4583 (1)	1648 (1)	2942 (0)	4590 (1)	9173 (2)
Mean (SD)	23.96 (3.444)	24.18 (3.460)	24.10 (3.455)	23.96 (3.303)	24.30 (3.607)	24.18 (3.504)	24.14 (3.480)
Median	23.60	23.90	23.80	23.70	24.20	24.00	23.90
Min, Max	16.3, 49.0	15.4, 42.8	15.4, 49.0	16.5, 49.9	14.1, 41.8	14.1, 49.9	14.1, 49.9
PCR results at baseline, n (%)							
Negative	1459 (89.2)	2412 (81.8)	3871 (84.4)	1497 (90.8)	2454 (83.4)	3951 (86.1)	7822 (85.3)
Positive	173 (10.6)	537 (18.2)	710 (15.5)	151 (9.2)	482 (16.4)	633 (13.8)	1343 (14.6)
Total (Missing)	1632 (3)	2949 (0)	4581 (3)	1648 (1)	2936 (6)	4584 (7)	9165 (10)
Vaccination intervals (months)							
N (Missing)	1635 (0)	2949 (0)	4584 (0)	1649 (0)	2942 (0)	4591 (0)	9175 (0)

	Placebo			SCTV01E			
	**<338 BAU/mL (N=1635)**	**≥338 BAU/mL (N=2949)**	**Total (N=4584)**	**<338 BAU/mL (N=1649)**	**≥338 BAU/mL (N=2942)**	**Total (N=4591)**	**All (N=9175)**
Mean (SD)	13.3 (2.93)	13.1 (2.95)	13.2 (2.95)	13.2 (2.98)	13.0 (2.98)	13.1 (2.98)	13.1 (2.96)
Median	13.0	13.0	13.0	13.0	13.0	13.0	13.0
Min, Max	6, 21	6, 24	6, 24	5, 24	6, 24	5, 24	5, 24

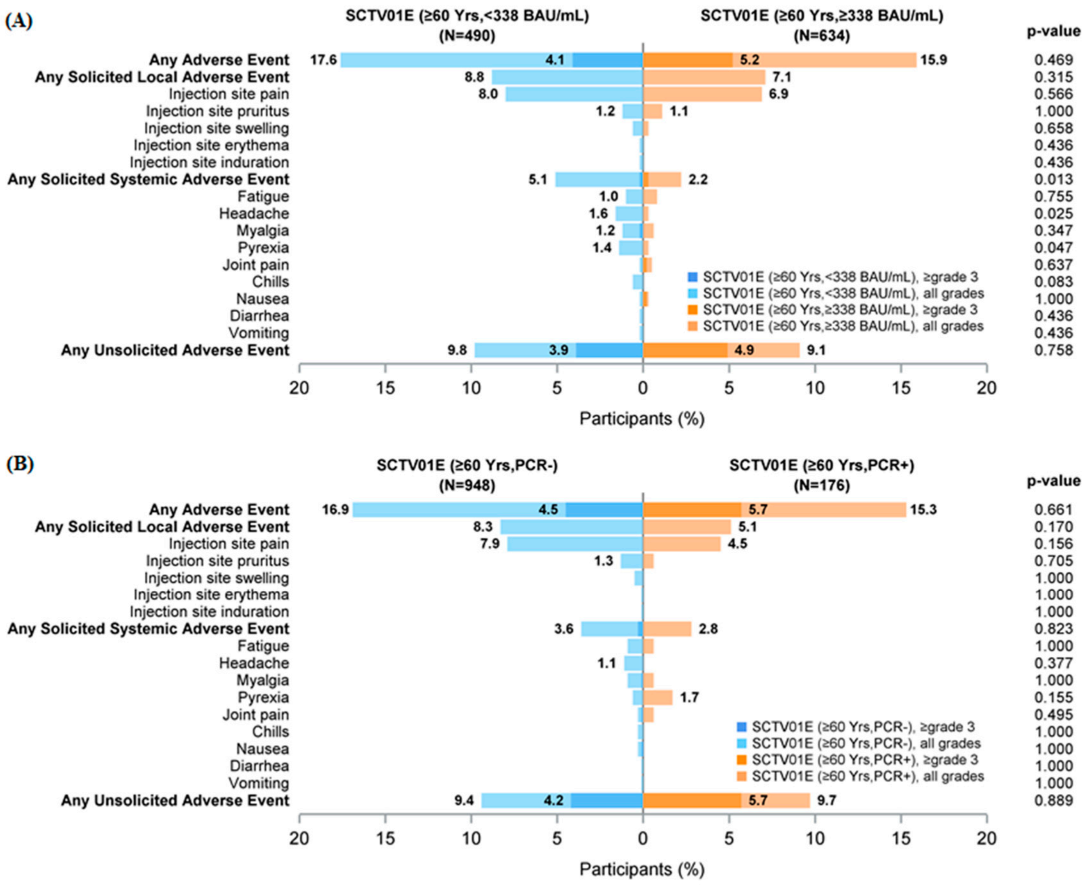

Figure S1. The incidence of adverse events is stratified by anti-SARS-CoV-2 IgG antibody levels and age (Panel A), PCR results and age (Panel B). Events classified as Grade ≥ 3 are defined as severe or higher-grade adverse events.
